# Effect of Thermal Processing on the Nutritional Composition and Bioactive Compounds of ‘Largueta’ Almonds (*Prunus dulcis*)

**DOI:** 10.3390/foods15122059

**Published:** 2026-06-07

**Authors:** María Fernanda Fernández-León, Luisel Rosana Flórez Acosta, Ana María Fernández-León

**Affiliations:** 1 Departmental Section of Food Technology, Veterinary Faculty, Complutense University of Madrid, Av. Puerta de Hierro S/N, 28040 Madrid, Spain; 2 Department of Postharvest, Plant, Value Enhancement and Emerging Technologies, Agrifood Technology Institute of Extremadura (INTAEX), Centre for Scientific and Technological Research of Extremadura (CICYTEX), Avda. Adolfo Suárez S/N, 06007 Badajoz, Spain

**Keywords:** almonds, blanching, roasting, oleic acid, amino acids, antioxidants

## Abstract

Almond (*Prunus dulcis*) is a highly nutritious nut, rich in unsaturated fatty acids, fibre, and bioactive compounds such as tocopherols, phenolic compounds, and carotenoids. Processing methods commonly applied in the almond industry, including blanching and roasting, may modify the nutritional composition and bioactive profile of the kernels. Therefore, the aim of this study was to evaluate the effect of blanching and roasting on the nutritional composition and bioactive compound content of the ‘Largueta’ almond variety. Three forms were analysed: raw almonds with skin, blanched (peeled) raw almonds, and roasted almonds, with their chemical composition, lipid profile and bioactive compound content being examined. The data obtained indicated that raw almonds with skin showed higher levels of fibre (12.16 g/100 g), phenolic compounds (66.35 mg gallic acid/100 g), and β-carotene (65.88 µg/100 g). Roasted almonds contained lower amounts of phenolic compounds (42.87 mg gallic acid/100 g), tocopherols (7.64 mg α-tocopherol/100 g and 1.99 mg γ-tocopherol/100 g) and essential amino acids such as tryptophan (1.23 g/100 g protein) and lysine (3.22 g/100 protein). Blanching, by removing the skin, significantly reduces fibre (7.52 g/100 g) and carotenes (26.60 µg β-carotene/100 g). With regard to fatty acids, the main components of nuts, oleic acid predominated in all samples (>65%), with no significant changes due to processing. Thermal treatments modify the composition of almonds. Roasting concentrates some nutrients but reduces antioxidants, while blanching mainly affects fibre content. Therefore, the consumption of raw sweet almonds with skin is recommended to preserve their nutritional and antioxidant benefits, or to subject them to moderate heat treatment.

## 1. Introduction

Almonds are among the most commercially important tree nuts worldwide due to their high production volume, economic relevance, nutritional value, and wide range of industrial applications. Global almond production in the current 2025/26 marketing year is forecast to rise by almost 10 per cent, reaching 1.8 million metric tonnes in shell, mainly due to increased production in the United States and, to a lesser extent, in the European Union and Australia. If realised, this would be the highest production level since the 2020/21 season [1].

In recent decades, almond consumption has increased considerably, driven by the growing consumer interest in healthy foods and plant-based diets. Almonds are recognised as an excellent source of protein, dietary fibre, unsaturated fatty acids, vitamins, and minerals, as well as bioactive compounds with antioxidant properties. Their regular consumption has been associated with several health benefits, including improvements in serum lipid profiles, glycaemic control, body weight regulation, and reduced risk of cardiovascular diseases, obesity, and type 2 diabetes [2].

The increasing global demand for almonds has also enhanced their economic importance in international markets. The United States remains the leading producer worldwide; however, Mediterranean countries, particularly Spain, play a major role in global almond production and trade. Spain is currently the world’s second-largest almond producer, and almond cultivation represents approximately 85% of the country’s total nut crop area. In the 2024/2025 season, Spanish almond production was 373,558 tonnes, representing an increase of 9.1% compared to the average production of the previous five seasons (2019–2023). Furthermore, almonds account for nearly 90% of the total value of nuts exported by Spain, with the European Union being the principal destination market, where Spanish almonds represent approximately 23% of market share, second only to almonds produced in the United States [3].

Due to their high nutritional value and commercial importance, almonds are consumed both raw and processed. Different processing technologies are applied to improve their sensory properties, shelf life, and suitability for industrial applications. Among the most common processing methods are roasting, blanching, particle size reduction, and oil extraction [4]. Thermal processing, particularly roasting and blanching, plays a critical role in determining the final quality characteristics of almonds, including texture, colour, flavour, and nutritional composition.

Roasting is one of the most widely used thermal treatments in the nut industry and involves the application of heat under controlled conditions to reduce moisture content and develop desirable sensory attributes. Depending on processing conditions such as temperature and time, roasted almonds can present light, medium, or dark colour intensities. Roasting improves crunchiness and promotes the development of characteristic roasted flavours through Maillard reactions and lipid-derived volatile compounds [5,6]. Previous studies have demonstrated that roasting significantly modifies almond physicochemical properties. For example, increases in hardness reduction and colour changes have been reported with increasing roasting temperatures, particularly reductions in lightness (L*) and increases in redness (a*) and yellowness (b*) values. In addition, roasting at elevated temperatures may increase the formation of volatile compounds such as pyrazines, furans, and aldehydes, which contribute to roasted aroma and flavour development. However, excessive temperatures can also promote lipid oxidation, degradation of heat-sensitive vitamins such as tocopherols, and loss of antioxidant activity, negatively affecting nutritional quality [5,6].

Temperature is considered one of the most critical factors during thermal processing because it directly influences both sensory quality and nutritional preservation. Moderate roasting temperatures may enhance flavour and texture while maintaining acceptable levels of bioactive compounds, whereas severe thermal conditions can accelerate oxidation reactions, reduce phenolic compounds, and alter fatty acid composition. Several studies have reported that roasting temperatures above 140–150 °C may significantly reduce antioxidant capacity and increase the formation of undesirable compounds associated with overheating. Therefore, optimising roasting conditions is essential to balance sensory improvement and nutritional preservation [7].

Blanching is another commonly applied thermal process in almond processing and is mainly used to remove the skin through wet or dry heating methods [7]. The efficiency of blanching depends on several factors, including almond variety, maturity stage, drying conditions, storage conditions, and irrigation practices during cultivation. Although blanching improves appearance and processing suitability, excessive thermal treatment may also affect almond texture, colour, flavour, and nutrient retention. Since almond skin contains a significant proportion of phenolic compounds and antioxidants, skin removal may reduce the antioxidant properties associated with almond consumption [8].

Considering the growing importance of almonds as antioxidant foods and the potential impact of thermal processing on their quality attributes, it is necessary to better understand how roasting and blanching conditions affect their nutritional composition and bioactive compounds. Therefore, the objective of this research was to evaluate the effect of thermal processing of ‘Largueta’ almonds, specifically roasting and blanching treatments, in order to determine how these processes influence their nutritional composition and health-related functional value.

## 2. Materials and Methods

### 2.1. Plan Material and Thermal Processing

The plant material used in this study consisted of almonds (*Prunus dulcis* Mill.) cv. ‘Largueta’ category I, characterised by their flat and elongated shape and widely used in the snack and roasted nut industry due to their easy peeling and good roasting aptitude. The almonds were obtained from a private traditional almond-growing farm located in Southeastern Extremadura, Spain, mainly dedicated to the production and export of almonds for nougat and snack manufacturing. Cultivation was carried out under organic farming practices, using natural products for soil and tree management. No information was available regarding the deposition of a voucher specimen, voucher number, or the herbarium/institution where such material was deposited.

It should be noted that the present study was conducted on the sweet almond cultivar ‘Largueta’, which contains negligible amounts of amygdalin compared with bitter almond varieties and is considered safe for consumption in its raw form under normal dietary conditions [2].

To remove the skin from the almonds, or peel them, the blanching process was used, immersing the almonds in water at 85–100 °C for about 3 min. The kernels were then dried at room temperature (22 ± 2 °C) for 30 min. However, roasting was carried out using hot air at temperatures of around 140 °C for approximately 15 min, followed by cooling (4 °C). After processing, all samples, raw almonds (RA), peeled almonds (PA) and roasted almonds (RTA), were packaged in multilayer pouches under a protective atmosphere consisting of 99% nitrogen and 1% residual oxygen using a vacuum-gas flushing system (Vapta Euvc-50, Hersill, Móstoles, Spain). The packaged samples were stored at 4 °C in the dark for up to 7 days prior to analysis.

### 2.2. Compositional Analysis

The methods used for compositional analysis were official methods of the International Association of Official Analytical Chemists [9]. The results were expressed in g or mg per 100 g of almonds. All analyses were carried out in triplicate to ensure reproducibility.

The energy content of the samples was theoretically estimated from their proximate composition using the Atwater general conversion factors. Energy values were calculated according to Equation (1), where protein, available carbohydrates, and lipid contents are expressed as g/100 g of sample. Conversion factors of 4 kcal/g were applied for proteins and available carbohydrates, whereas 9 kcal/g was used for lipids, according to the Atwater general factors.

Energy (kcal/100 g) = (4 × Protein) + (4 × Available Carbohydrates) + (9 × Lipids)(1)

Total carbohydrates were determined by difference according to Equation (2), where all components are expressed as g/100 g of sample. The calculation was performed based on the proximate composition obtained for each sample.

Total carbohydrates (g/100 g) = 100 − (Moisture + Protein + Lipids + Ash)(2)

Sugar analysis was performed by high-performance liquid chromatography (HPLC) using an Agilent Technologies HPLC system (Santa Clara, CA, USA) equipped with a refractive index detector (RID). Separation was achieved using a carbohydrate analysis column maintained at controlled temperature conditions. The mobile phase consisted of acetonitrile/water (75:25, *v*/*v*), delivered under isocratic conditions at a flow rate of 1.0 mL/min. Sugars were identified and quantified by comparison of retention times and peak areas with those obtained from external standards of glucose, fructose, sucrose, and maltose prepared at known concentrations; the results were expressed as g/100 g of sample.

Total dietary fibre was determined using the enzymatic–gravimetric method. Samples were sequentially digested with thermostable α-amylase, protease and amyloglucosidase. The residue was precipitated with ethanol, filtered, dried and corrected for protein and ash content.

Protein content was determined using the Kjeldahl method using a digestion unit and distillation system (Kjeltec 2200, FOSS, Hillerød, Denmark). Total nitrogen was quantified after acid digestion, distillation and titration, and protein content was calculated using a nitrogen-to-protein conversion factor of 6.25.

Salt content was estimated indirectly from sodium concentration determined according to AOAC official methods using atomic absorption spectrophotometry (AAS). Prior to analysis, samples were subjected to acid digestion using nitric acid under controlled heating conditions to obtain complete mineral extraction. Sodium determination was carried out using an atomic absorption spectrophotometer (3100 Perkin Elmer, Waltham, MA, USA) under the instrumental conditions recommended by the manufacturer. Quantification was performed by external calibration using sodium standard solutions of known concentration. Sodium chloride content was subsequently calculated from the measured sodium concentration using the stoichiometric conversion factor of 2.5 according to Equation (3). Results were expressed as g NaCl/100 g of sample.

NaCl (g/100 g) = Na (g/100 g) × 2.5(3)

Phosphorus determination was carried out after acid digestion of the samples using a colorimetric spectrophotometric method based on the formation of a phosphomolybdate complex. The resulting coloured complex was measured at 880 nm using a Jenway™ 6305 UV/visible spectrophotometer (Jenway, Stone, Staffordshire, UK). Calibration curves were established using certified phosphorus standard solutions prepared at different concentration levels, and quantification was performed by external calibration.

Calcium and magnesium contents were determined after acid digestion of the samples using a ContrAA 700 high-resolution continuum source atomic absorption spectrometer (Analytik Jena, Turingia, Germany). Calcium and magnesium were quantified at wavelengths of 422.7 nm and 285.2 nm, respectively, under the instrumental conditions recommended by the manufacturer. Calibration curves were prepared using certified standard solutions at different concentration levels, and quantification was performed by external calibration. Quality control standards and reagent blanks were included throughout the analytical procedure to verify analytical accuracy and instrument stability.

### 2.3. Determination of Fatty Acid Content (FAs)

Total lipids were extracted from almond samples using a chloroform/methanol mixture following standard procedures. Fatty acid methyl esters (FAMEs) were obtained by transesterification according to the method described in BOE No. 283 [10]. The separation, identification, and quantification of fatty acids were performed by gas chromatography using a KNK-3000-HRGC gas chromatograph (Konik Instruments, Barcelona, Spain) equipped with a flame ionisation detector (FID). Fatty acid methyl esters (FAMEs) were separated using a capillary GC column (BPX70 cyanopropyl polysilphenylene–siloxane stationary phase column; 30 m × 0.25 mm i.d., 0.25 μm film thickness). Helium was used as the carrier gas at a constant flow rate of 1.0 mL/min. The oven temperature programme was as follows: initial temperature of 140 °C held for 5 min, increased at 4 °C/min to 220 °C, and maintained at the final temperature for 10 min. Injector and FID temperatures were set at 250 °C and 260 °C, respectively. Samples were injected in split mode under the chromatographic conditions recommended for FAME analysis. Individual fatty acids were identified by comparing their retention times with those of certified FAME external standards analysed under identical conditions. Quantification was carried out by peak area normalisation and expressed as g/100 g of almonds. All analyses were performed in triplicate to ensure analytical reproducibility.

### 2.4. Determination of Amino Acid Content

Amino acid composition was determined according to the method described by Henderson et al. (2000) [11]. Samples were subjected to acid hydrolysis, and the released amino acids were derivatised using o-phthalaldehyde (OPA) for primary amino acids and 9-fluorenylmethyl chloroformate (FMOC) for secondary amino acids. Derivatised amino acids were separated and analysed by high-performance liquid chromatography (HPLC) using an Agilent 1100 HPLC system equipped with a diode array detector (DAD) (Agilent Technologies, California, USA). Chromatographic separation was achieved using a ZORBAX Eclipse AAA analytical column (4.6 mm × 150 mm, 5 μm particle size; Agilent Technologies, USA), specifically designed for amino acid analysis following OPA/FMOC derivatisation. Detection was performed at 338 nm for OPA derivatives and at 262 nm for FMOC derivatives. Individual amino acids were identified by comparison of retention times and UV spectra with those of certified external standards analysed under identical chromatographic conditions. Quantification was carried out using external calibration curves prepared from amino acid standard solutions of known concentrations. Results were expressed as g amino acid/100 g protein. All analyses were performed in triplicate to ensure analytical reproducibility.

### 2.5. Determination of Total Phenolic Compounds

Phenolic compounds were extracted from almond samples following a standardised procedure. Approximately 2 g of almonds were first chopped and homogenised with 25 mL of 70% (*v*/*v*) ethanol using a mechanical homogeniser (Ika Ultra-Turrax, Staufen im Breisgau, Germany) to ensure complete disruption of the tissue. The homogenates were centrifuged at 5000× *g* for 15 min at 4 °C using a refrigerated centrifuge (5810R, Eppendorf, Hamburgo, Germany), and the resulting supernatants were carefully collected for subsequent analysis. Total phenolic content was determined using the Folin–Ciocalteu colorimetric assay, following the method described by Singleton and Rossi (1965) [12]. Briefly, an aliquot of the extract was mixed with the Folin–Ciocalteu reagent and, after a reaction period, a 20% sodium carbonate solution was added to develop the colour. The mixture was incubated under controlled conditions, and absorbance was measured at 760 nm using a spectrophotometer (Jenway™ 6305 UV/visible spectrophotometer, Jenway, Stone, Staffordshire, UK). Quantification was performed using a calibration curve prepared with gallic acid as the external standard, and results were expressed as mg of gallic acid equivalents (GAE) per 100 g of almonds. All analyses were carried out in triplicate to ensure reproducibility.

### 2.6. Determination of Tocopherols

Alpha (α) and gamma (γ) tocopherols were extracted and quantified following the method proposed by Liu et al. (1996) [13]. Briefly, samples were homogenised and saponified under controlled conditions to release the tocopherols, followed by extraction with isooctane. Tocopherol analysis was performed using an Agilent 1100 Series HPLC system (Agilent Technologies, California, USA) equipped with a fluorescence detector (FLD). The detector was operated at an excitation wavelength of 295 nm and an emission wavelength of 330 nm for tocopherol detection. Separation was achieved using a reversed-phase C18 analytical column (ZORBAX Eclipse XDB-C18, 4.6 mm × 150 mm, 5 μm particle size; Agilent Technologies, USA). The mobile phase consisted of methanol (isocratic elution) at a flow rate of 1.0 mL/min, and the column temperature was maintained at 25 °C. Individual tocopherols were identified by comparison of retention times with those of authentic α- and γ-tocopherol standards analysed under identical conditions. Quantification was performed using external calibration curves constructed with certified standards. Results were expressed as mg of α- or γ-tocopherol per 100 g of almonds. All analyses were performed in triplicate to ensure reproducibility.

### 2.7. Determination of Carotenoids

Carotenoid content was determined following the method described by Nagata and Yamashita (1992) [14]. About 1 g of almond was homogenised and the pigments extracted using 10 mL of an acetone–hexane mixture (4:6, *v*/*v*). The extracts were filtered, and the absorbance of the resulting solution was measured at 663, 645, 505, and 453 nm using a Jenway™ 6305 UV/visible spectrophotometer (Jenway, Stone, Staffordshire, UK). β-carotene concentration was calculated using the following Equation (4), where A_663_, A_645_, A_505_, and A_453_ correspond to the absorbance values measured at the respective wavelengths.

β-carotene (mg/100 mL) = 0.216A_663_ − 1.22A_645_ − 0.304A_505_ + 0.452A_453_ (4)

The results were expressed as µg of β-carotene per 100 g of almonds. All measurements were performed in triplicate to ensure reproducibility.

### 2.8. Statistics

SPSS 15.0 (SPSS Inc., Chicago, IL, USA) was used for statistical analyses. Data were expressed as mean ± standard deviation (SD) from independent analyses and samples. Mean values were compared using Student’s *t*-test with the significance level set at α = 0.05. All analyses were performed in triplicate to ensure reproducibility.

Principal component analysis (PCA) was performed using Microsoft Excel v. 2603 to evaluate relationships among samples and variables. Sample scores and variable loadings were calculated, and a two-dimensional biplot based on the first two principal components was generated to visualise sample distribution and variable contributions. All analyses were carried out using Microsoft Excel (Microsoft Corp., Redmond, WA, USA).

## 3. Results and Discussion

### 3.1. Energetic and Nutritional Values

Table 1 shows the average energetic and nutritional values per 100 g of almonds in their three analysed forms, RA, PA and RTA.

No significant differences were observed in the energy value among raw almonds (RA, 596.12 kcal/100 g), peeled almonds (PA, 617.04 kcal/100 g), and roasted almonds (RTA, 608.10 kcal/100 g). The relatively stable caloric content suggests that the processing treatments applied did not substantially alter the major energy-contributing macronutrients, particularly lipids, which represent the primary source of energy in almonds. Although roasting promotes moisture loss and may increase the relative concentration of nutrients on a weight basis, the magnitude of these changes was insufficient to produce significant differences in energy value [7]. Furthermore, while thermal processing can induce lipid oxidation and Maillard reactions involving proteins and carbohydrates, these reactions generally affect only a small proportion of the total macronutrient content and therefore have a limited impact on overall caloric value. Similar observations have been reported in studies on roasted tree nuts, where changes in nutrient composition following processing did not result in substantial alterations in energy density [15]. The slightly higher energy value observed in peeled almonds may be related to the relative concentration of fat and protein fractions following the removal of the fibre-rich skin, although these differences were not statistically significant.

Carbohydrate content was significantly reduced in peeled almonds (PA; 3.54 ± 0.51 g/100 g) compared with raw almonds (RA; 6.80 ± 0.91 g/100 g) and roasted almonds (RTA; 4.79 ± 0.32 g/100 g) (*p* < 0.05). Similarly, sugar content was higher in RA (3.92 ± 0.14 g/100 g) than in the processed samples. These findings are consistent with those reported by Folasade and Subomi (2016) [15], who also observed reductions in sugar content after roasting. The decrease in sugars may be explained by their participation in Maillard reactions, in which reducing sugars react with free amino groups of proteins and amino acids, leading to the formation of melanoidins and other browning products. In addition, thermal degradation and caramelisation reactions may further contribute to the depletion of simple sugars during roasting. The extent of these reactions depends on processing temperature, duration, and the availability of reactants, and they are known to influence both the nutritional and sensory characteristics of nuts [16].

Dietary fibre content was significantly lower in peeled almonds (7.52 ± 0.43 g/100 g) than in raw (12.16 ± 0.84 g/100 g) and roasted almonds (11.04 ± 0.50 g/100 g), which showed statistically similar values. These results indicate that almond skin is a major source of dietary fibre and that its removal substantially decreases total fibre content. Almond skins contain considerable amounts of insoluble polysaccharides, including cellulose, hemicellulose, and lignin, which account for a large proportion of total dietary fibre. The absence of significant differences between raw and roasted almonds suggests that the thermal treatment applied was not sufficiently severe to promote extensive degradation of structural polysaccharides. In contrast, Folasade and Subomi (2016) [15] reported increased fibre levels after heat treatment, which may be attributed to differences in processing conditions, botanical origin, or analytical methodologies. Furthermore, thermal processing can increase the relative proportion of fibre by reducing moisture content and concentrating dry matter components.

Protein content differed significantly among samples, ranging from 24 to 27 g/100 g. These results agree with previous studies [15] reporting a slight reduction in protein levels after roasting. Heat treatment can induce protein denaturation, altering protein structure and exposing reactive amino acid residues. This process may facilitate Maillard reactions between amino groups and reducing sugars, resulting in the formation of high-molecular-weight complexes and a reduction in the availability of certain essential amino acids, particularly lysine. In addition, thermal processing may decrease the digestibility and nutritional quality of proteins when excessive temperatures promote the formation of advanced glycation end-products [7,16]. Nevertheless, the relatively small differences observed suggest that the roasting conditions employed in the present study preserved most of the almond protein fraction.

No significant differences were detected in salt content, which remained constant at 0.03 g/100 g in all samples. This stability is expected because sodium is a mineral element that is generally resistant to thermal degradation under conventional food-processing conditions [17]. Therefore, the observed values likely reflect the natural sodium content of almonds rather than any effect of processing.

Regarding mineral composition, phosphorus content was slightly lower in peeled almonds (505.22 ± 6.50 mg/100 g), whereas calcium and magnesium concentrations remained stable among treatments. The reduction in phosphorus may be related to the removal of the skin and outer layers of the kernel, where part of the mineral fraction is concentrated, as well as to potential leaching losses during blanching. In contrast, calcium and magnesium appeared less affected by the processing conditions applied. These findings differ from those reported by Massantini and Frangipane (2022) [17], who observed increases in phosphorus, calcium, and magnesium contents following roasting. Such increases are often attributed to moisture loss during heating, which concentrates nutrients on a dry-weight basis, and to the degradation of antinutritional compounds such as phytic acid, thereby increasing mineral extractability. The stability of calcium and magnesium observed in the present study suggests that the mild roasting treatment used did not substantially modify mineral retention or availability.

### 3.2. Fatty Acid Content

The values are shown in Table 2, and the data will be discussed in groups.

#### 3.2.1. Saturated Fatty Acids (SFA)

The values obtained for palmitic acid (C16:0), which is one of the main saturated fatty acids present in almonds, were similar among the three treatments (6.84 ± 0.17 g/100 g in raw almonds, 6.88 ± 0.04 g/100 g in raw peeled almonds and 6.86 ± 0.01 g/100 g in roasted almonds), with no statistically significant differences. This indicates that neither peeling nor roasting affects the concentration of this fatty acid, suggesting its thermal stability during processing. In contrast, Oliveira et al. (2020) [18] reported a general tendency for this fatty acid to decrease after processing, although a slight increase was observed in some cultivars. This difference may be due to both the genetic diversity of the cultivars used and the more intense blanching and roasting conditions used, which could have induced greater changes in the lipid fraction.

The levels of stearic acid (C18:0) were affected by processing. While the raw almond had a higher value (3.22 ± 0.01 g/100 g), both the peeled (3.01 ± 0.05 g/100 g) and roasted (3.00 ± 0.02 g/100 g) almonds showed significant reductions, suggesting that skin removal and heat treatment may induce slight losses of this fatty acid, possibly through oxidation or lipid redistribution. This is consistent with the results of other authors [18,19], who also reported a reduction in most of the cultivars analysed.

The arachidic acid (C20:0) content ranges from 0.11 to 0.13 g/100 g in the three treatments, with no significant differences. This result differs from others [20], who observed a significant increase in total saturated fatty acids in almonds after frying, while roasting produced a more moderate increase. This difference can be attributed to the type of thermal processing, with frying being a more aggressive treatment that could induce changes in lipid composition due to the absorption of cooking oil. In this regard, the data from this study suggest that roasting, under the conditions applied in this study, does not affect the arachidic acid content in almonds.

#### 3.2.2. Monounsaturated Fatty Acids (MUFA)

Palmitoleic acid (C16:1) showed a slight decrease in RTA (0.56 ± 0.02 g/100 g) compared to RA (0.65 ± 0.02 g/100 g) and PA (0.63 ± 0.001 g/100 g), this difference being significant. This decrease may be related to the susceptibility of palmitoleic acid to oxidation or thermal degradation during roasting, a process in which temperatures close to 140 °C are applied, as has been observed in other MUFA-rich oils [21].

Oleic acid (C18:1n9c) is the predominant fatty acid in almonds, accounting for 69–70% of the total lipid content. No significant differences were observed between the samples (69.33 ± 0.16 g/100 g in RA, 70.08 ± 0.03 g/100 g in PA and 69.47 ± 0.06 g/100 g in RTA), indicating that both peeling and roasting do not affect the concentration. This indicates high heat stability and is consistent with studies highlighting the resistance of oleic acid to moderate oxidation, such as that by Shahidi and Zhong (2010) [21]. The stability of oleic acid is a positive characteristic, as this compound is associated with cardiovascular benefits and resistance to oxidation. Oleic acid represents the largest proportion of fat in almonds (around 70% of total fat content), highlighting its importance as a source of omega-9.

The values for gadoleic acid (C20:1n11) are very consistent, around 0.08 to 0.09 g/100 g, with no notable statistical differences between raw, peeled, or roasted almonds. This indicates the stability of this fatty acid when subjected to the treatments applied [21].

#### 3.2.3. Polyunsaturated Fatty Acids (PUFA)

Linoleic acid (C18:2n6t) is the second most abundant fatty acid (almost 20%). No significant differences were detected, which can be attributed to this compound’s resistance to moderate thermal processes, as observed in studies on nuts and oils [20].

Linolenic acid (C18:3n6) is a minor component that remained constant in all samples (values between 0.04 and 0.05 g/100 g), with no significant differences. This shows that the treatment applied does not significantly affect this minor component. However, divergent results have been reported by Oliveira et al. (2020) [18], who observed statistically significant differences after subjecting different varieties of almonds to roasting and blanching processes. This discrepancy could be attributed to varietal differences, temperatures, processing times used, as well as methodological sensitivity in the quantification of minor components.

Both fatty acids are omega-6, which helps reduce LDL cholesterol, improve cardiovascular health, play a structural role in cell membranes, and are precursors to eicosanoids, compounds that regulate inflammatory and immune processes. It should be noted that almonds are not a significant source of omega-3, so it is important to consume other sources of omega-3 to maintain the omega-6/omega-3 ratio [22].

**Figure 1:**
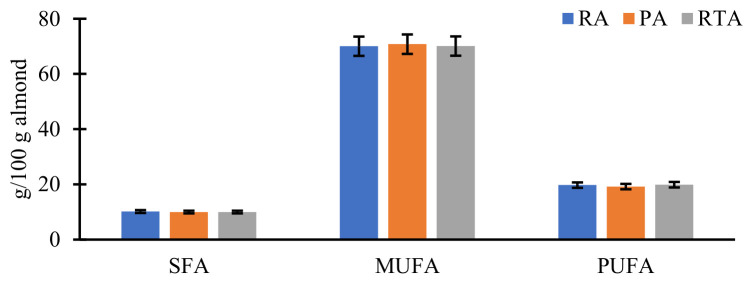
Saturated fatty acid (SFA), monounsaturated fatty acid (MUFA) and polyunsaturated fatty acid (PUFA) content in raw (RA), peeled (PA) and roasted (RTA) almonds.

Furthermore, as shown in Figure 1, almonds have a highly favourable lipid profile from a nutritional point of view, with a predominance of monounsaturated fatty acids (MUFA ~70%) and a significant proportion of polyunsaturated fatty acids (PUFA ~20%). Peeling and roasting do not significantly affect these contents. The results are consistent with those reported in the scientific literature [19], since the statistical analysis revealed no significant differences (*p* > 0.05) in SFA, MUFA or PUFA contents, indicating that the treatments did not significantly alter the fatty acid composition of almonds.

### 3.3. Amino Acids

Heat treatment applied to almonds produced a decrease in the relative concentration of most amino acids (Table 3), with more pronounced reductions in the more intense treatment, such as roasting. However, statistically significant decreases only occurred in roasted almonds for the essential amino acids tryptophan, lysine, and methionine + cysteine (reductions of approximately −11.5%, −8.0%, and −5.4%, respectively). Meanwhile, the most abundant and non-essential amino acids, such as aspartic acid and glutamic acid, experienced minimal variations (approximately −3% and −2.5%, respectively). This pattern is consistent with the literature on heat-processed almonds [23], where the Maillard reaction, oxidation and thermal degradation explain preferential reductions in labile or highly reactive amino acids, especially lysine, whose ε-amino group is particularly susceptible to glycation.

Although the absolute losses are relatively moderate, they are significant from a nutritional perspective, given that lysine and sulphur amino acids are already considered limiting factors in almond protein. Therefore, their reduction after roasting could negatively affect protein quality indicators [2,24]. In addition, the presence of precursors and roasting conditions (temperature/time) facilitates the formation of acrylamide in roasted almonds; the literature recommends controlling the roasting temperature (e.g., keeping it below ~130–150 °C to minimise acrylamide, conditions such as ours) when seeking to preserve nutritional quality and safety. This becomes especially important if heat treatments are intense [25].

### 3.4. Total Phenolic Compound Content

**Figure 2:**
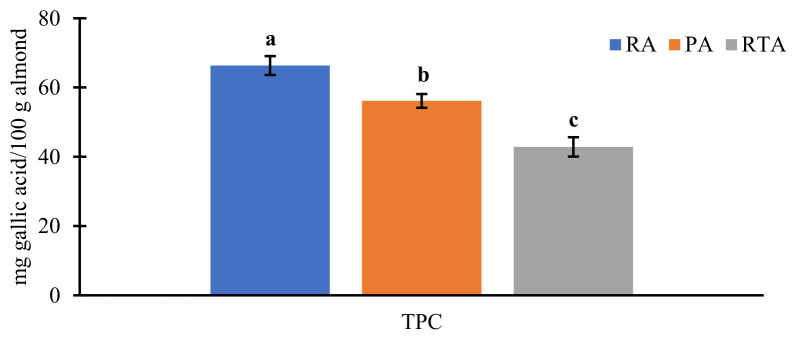
Total phenolic compound content (TPC) in raw (RA), peeled (PA) and roasted (RTA) almonds. Different letters indicate that there are significant differences between the values (*p* ≤ 0.05).

Raw almonds had the highest phenolic compound content (66.35 mg gallic acid/100 g), confirming that these compounds are mainly concentrated in the skin of the fruit. Secondly, the highest concentration was observed in raw peeled almonds (56.14 mg gallic acid/100 g) and, finally, the lowest value (42.87 mg gallic acid/100 g) was obtained in roasted almonds (Figure 2).

These findings do not coincide with the results obtained by Ghazzawi and Al-Ismail (2017) [20], who also evaluated the effect of roasting on phenolic compounds in almonds. In their study, no significant differences in total phenolic content were observed between raw and roasted almonds, which could be attributed to differences in roasting temperature (110 °C for 16 min in their case) and analysis methodology. However, their study suggests that in some nuts, such as pine nuts, roasting can increase phenolic content due to the formation of Maillard reaction products that react with the Folin–Ciocalteu reagent and could overestimate total phenols.

In this study, the significant reduction in phenolic content after roasting suggests thermal degradation of the polyphenols present, especially in the almond skin, where most of these compounds are concentrated. It is possible that the roasting conditions used exceeded the thermal stability threshold of certain phenols, promoting their oxidation or destruction. On the other hand, the blanched almond (PA) had an intermediate concentration, which shows that blanching removes part of these compounds by removing the skin, but with less thermal impact than roasting, thus preserving a higher proportion of polyphenols.

These findings highlight the importance of the processing method in preserving the bioactive compounds in almonds. While blanching reduces phenolic content by physically removing the skin, roasting directly affects the stability of polyphenols through the action of heat, even in the inner part of the fruit. This suggests that, from a nutritional and bioactive point of view, consuming raw or minimally processed almonds could be more beneficial if the aim is to preserve the natural antioxidant content as much as possible.

### 3.5. Tocopherols

**Figure 3:**
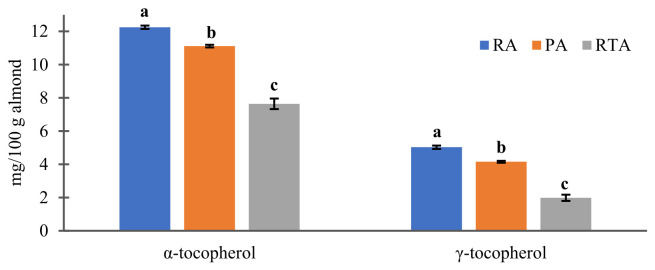
Tocopherols content in raw (RA), peeled (PA) and roasted (RTA) almonds. The Different letters indicate that there are significant differences between the values (*p* ≤ 0.05).

The results reveal that the α-tocopherol content was significantly higher in raw almonds, with a decrease after peeling and roasting treatments. Raw almonds reached the highest levels (12.24 mg/100 g), followed by peeled almonds (11.11 mg/100 g), while roasted almonds had the lowest content (7.64 mg/100 g) of this compound (*p* ≤ 0.05) (Figure 3).

A similar pattern was observed for γ-tocopherol. Raw almonds had the highest content (5.0.3 mg/100 g), followed by peeled almonds (4.15 mg/100 g), and roasted almonds had the lowest value (1.99 mg/100 g) (Figure 3). These results are consistent with those reported by other authors [26], who reported a decrease of up to 63% in α-tocopherol after roasting almonds at 165 °C for 15 min, as well as losses of close to 20% in γ-tocopherol at temperatures of 140–165 °C. The degradation observed can be attributed to the thermal sensitivity of these lipophilic compounds, which are susceptible to heat-induced oxidation and exposure to oxygen. In the case of blanching prior to peeling, the losses were lower, suggesting that even brief and mild heat treatments can partially compromise the stability of tocopherols.

From a nutritional perspective, these results have significant implications. α-Tocopherol is the most active form of vitamin E in humans, and its preservation in food represents added value for the diet. The fact that raw almonds retain higher concentrations of tocopherols suggests that, to maximise their antioxidant value, it is preferable to consume them without heat treatment [27].

### 3.6. β-Carotene

**Figure 4:**
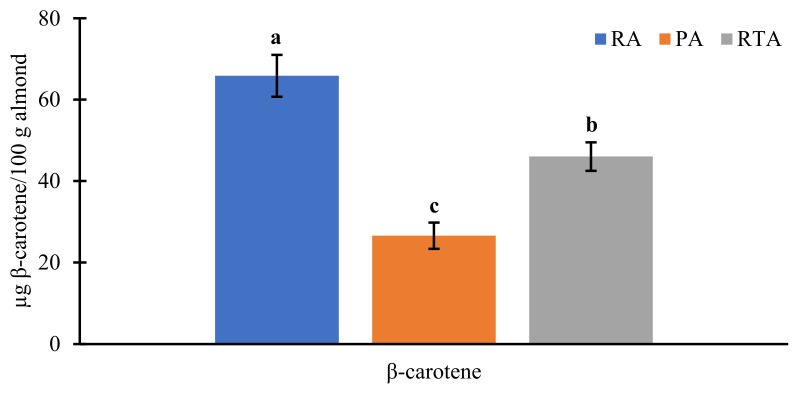
β-carotene content in raw (RA), peeled (PA) and roasted (RTA) almonds. Different letters indicate that there are significant differences between the values (*p* ≤ 0.05).

The results obtained in this study (Figure 4) show that the β-carotene content in raw almonds (65.88 µg/100 g) is significantly higher than that found in almonds subjected to heat treatment, especially after blanching to remove the skin, where a considerable reduction to 26.60 µg/100 g was observed. Roasted almonds had an intermediate concentration (46.04 µg/100 g), indicating a negative impact of heat treatment, but less drastic than blanching. These findings partially coincide with those reported in previous studies [27], which found that roasting at 160–170 °C significantly reduces β-carotene in almonds, with an approximate reduction of 50%. Although the magnitude of the reduction in our study is smaller, probably due to differences in specific roasting conditions and analysis methods, the trend towards a decrease in β-carotene with heat treatments is consistent.

### 3.7. Principal Component Analysis (PCA)

**Figure 5:**
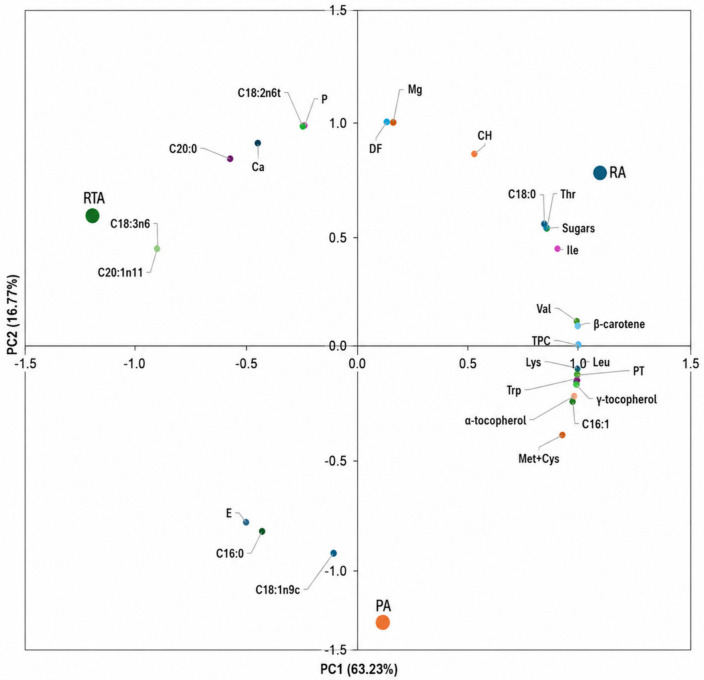
Principal component analysis (PCA) biplot showing the discrimination of raw almonds (RA), peeled almonds (PA), and roasted almonds (RTA) based on nutritional and bioactive composition, including energetic value (E), carbohydrates (CH), sugars, dietary fibre (DF), proteins (PT), minerals (P, Ca and Mg), fatty acids (C16:0, C18:0, C20:0, C16:1, C18:1n9c, C20:1n11, C18:2n6t and C18:3n6), amino acids (Ile, Leu, Lys, Met + Cys, Phe + Tyr, Thr, Trp and Val), total phenolic compound content (TPC), α-tocopherol, γ-tocopherol and β-carotene.

Principal component analysis (PCA) was applied to evaluate the effect of processing (raw, peeled and roasted) on the nutritional, lipidic, mineral, amino acid and antioxidant composition of almonds (Figure 5). The first two principal components explained 79.99% of the total variance, with PC1 accounting for 63.23% and PC2 for 16.77%, indicating that the bidimensional model adequately summarised the multivariate structure of the dataset. In food matrices, cumulative variance above 70% in the first two components is generally considered satisfactory for biological interpretation [28].

The PCA score plot revealed a clear discrimination among raw almonds (RA), peeled almonds (PA) and roasted almonds (RTA), demonstrating that postharvest processing significantly modified almond composition. Similar findings have been reported in almond kernels and other tree nuts, where roasting and skin removal were identified as major drivers of chemical variability [29,30].

The first component (PC1) appeared to represent the main gradient associated with nutritional density and bioactive properties. Positive PC1 values were closely associated with carbohydrates, sugars, proteins, essential amino acids (Leu, Ile, Lys, Thr, Trp and Val), total phenolic compounds (TPC), α-tocopherol, γ-tocopherol and β-carotene. This distribution suggests that samples located in the positive region of PC1 presented enhanced nutritional and antioxidant characteristics.

In contrast, negative PC1 values were associated with calcium, phosphorus and several minor fatty acids, including C20:0, C18:2n6t, C18:3n6 and C20:1n11. This pattern may indicate an opposition between metabolically available nutrients and more structural fractions of the almond kernel, such as mineral-associated tissues and specific lipid components. Comparable nutrient contrasts have previously been described in multivariate analyses of plant foods and oilseeds [31].

The second component (PC2), which explained 16.77% of the total variance, represented an additional independent source of variation strongly related to processing effects. This axis likely reflects modifications induced by roasting and skin removal, including moisture loss, concentration effects, redistribution of lipid fractions, disruption of cellular structure, changes in antioxidant extractability, and alterations in protein conformation.

The relatively high contribution of PC2 is likely associated with modifications in the physicochemical organisation of the almond matrix. Thermal processing is known to modify nut microstructure and lipid accessibility while promoting Maillard reaction pathways and degradation of heat-sensitive compounds [32].

Raw almonds (RA) were located in the positive region of PC1, in close association with sugars and several nutrient-related variables, suggesting a compositional profile characteristic of unprocessed almonds. Raw kernels generally maintain intact cellular structures and naturally high levels of tocopherols, especially α-tocopherol, the predominant vitamin E isomer in almonds [33]. Their PCA position suggests a balanced nutritional profile.

Peeled almonds (PA) were clearly separated from raw almonds, confirming that skin removal significantly modified composition. Almond skin is recognised as an important source of polyphenols, flavonoids, condensed tannins and dietary fibre, and contributes substantially to the antioxidant capacity of the whole kernel [29]. Therefore, the displacement of PA away from antioxidant-related variables suggests a reduction in bioactive compounds after peeling. Although peeling improves texture, appearance and sensory acceptance, it commonly reduces the concentration of health-promoting phytochemicals.

Roasted almonds (RTA) occupied a distinct region of the PCA plot, confirming that roasting induced marked compositional changes. Roasting is known to promote the formation of volatile aroma compounds, Maillard reaction products, increased oil extractability, structural softening, and partial degradation of thermolabile antioxidants. It may also affect amino acid reactivity, particularly lysine availability under intense heat conditions [32]. The clear separation of RTA indicates that thermal treatment substantially modified the chemical fingerprint of almond kernels.

The close grouping of Leu, Ile, Val, Lys, Thr and Trp indicates a strong positive correlation among essential amino acids, probably due to their common origin in almond storage proteins. Likewise, the clustering of TPC, α-tocopherol, γ-tocopherol and β-carotene suggests coordinated antioxidant behaviour, indicating that samples richer in one antioxidant fraction also tended to be richer in others.

In addition, the proximity among Ca, P and Mg suggests common accumulation patterns linked to structural tissues and mineral distribution within the kernel.

The PCA demonstrated that processing was the main factor driving compositional variability in almonds. Peeling mainly reduced skin-associated antioxidants and fibre, whereas roasting induced broader changes affecting lipids, antioxidants and matrix structure. Raw almonds retained a profile closer to the unprocessed kernel composition.

## 4. Conclusions

Blanching (PA) significantly reduced carbohydrate and dietary fibre contents, most likely due to the removal of the skin, which is rich in structural polysaccharides. Roasting (RTA) caused a slight decrease in protein content and reduced some essential amino acids sensitive to heat or Maillard reactions, particularly lysine, methionine + cysteine, and tryptophan. Non-essential amino acids showed only minor changes, while peeling had little effect on the overall amino acid composition of the ‘Largueta’ almond.

Mineral composition (P, Ca, and Mg) remained stable after both peeling and roasting, indicating that these treatments do not substantially affect the mineral profile. Likewise, the lipid fraction showed high stability. Only small reductions were detected in stearic acid (C18:0) after PA and RTA, and in palmitoleic acid (C16:1) after roasting. No significant differences were observed in the major fatty acids, oleic and linoleic acids, which together represented more than 85% of the total fatty acids. These results confirm the strong resistance of the lipid profile to moderate thermal processing.

Raw almonds (RA) presented the highest total phenolic content (TPC), significantly exceeding PA and RTA samples. Peeling reduced TPC, confirming that the skin is a major source of phenolic compounds, while roasting caused the greatest losses, likely due to oxidation and thermal degradation. A similar pattern was found for β-carotene: RA showed the highest values, PA the lowest, and RTA intermediate levels.

Overall, the Largueta almond variety exhibited high nutritional and bioactive stability under moderate processing conditions, supporting its suitability for snack production and industrial use.

## Figures and Tables

**Table 1:** Energetic and nutritional values per 100 g of almonds in their three analysed forms: raw almonds (RA), peeled almonds (PA) and roasted almonds (RTA).

	RA	PA	RTA
Energetic value (kcal)	596.12 ± 12.10 ^a^	617.04 ± 14.09 ^a^	608.10 ± 10.05 ^a^
Carbohydrates (g)	6.80 ± 0.91 ^a^	3.54 ± 0.51 ^c^	4.79 ± 0.32 ^b^
Sugars (g)	3.92 ± 0.14 ^a^	2.61 ± 0.11 ^b^	2.51 ± 0.20 ^b^
Dietary fibre (g)	12.16 ± 0.84 ^a^	7.52 ± 0.43 ^b^	11.04 ± 0.50 ^a^
Proteins (g)	27.05 ± 0.55 ^a^	26.12 ± 0.91 ^ab^	24.42 ± 0.80 ^b^
Salt (g)	0.03 ± 0.00 ^a^	0.03 ± 0.00 ^a^	0.03 ± 0.00 ^a^
Phosphorus (mg)	525.14 ± 9.22 ^a^	505.22 ± 6.50 ^b^	529.05 ± 7.34 ^a^
Calcium (mg)	296.28 ± 2.95 ^a^	291.15 ± 3.74 ^ab^	299.06 ± 3.50 ^a^
Magnesium (mg)	250.06 ± 3.51 ^a^	246.26 ± 2.90 ^a^	249.43 ± 4.84 ^a^

The salt content is exclusively due to naturally occurring sodium. Mean value ± standard deviation. The same letter in the same row indicates that there are no significant differences between the values (*p* > 0.05).

**Table 2:** Fatty acid content per 100 g of almonds in their three analysed forms: raw almonds (RA), peeled almonds (PA) and roasted almonds (RTA).

	Fatty Acids (g/100 g Almond)	RA	PA	RTA
SFA	C16:0	6.84 ± 0.17 ^a^	6.88 ± 0.04 ^a^	6.86 ± 0.01 ^a^
	C18:0	3.22 ± 0.01 ^a^	3.01 ± 0.05 ^b^	3.00 ± 0.02 ^b^
	C20:0	0.12 ± 0.00 ^a^	0.11 ± 0.01 ^a^	0.13 ± 0.00 ^a^
MUFA	C16:1	0.65 ± 0.02 ^a^	0.63 ± 0.00 ^a^	0.56 ± 0.02 ^b^
	C18:1n9c	69.33 ± 0.16 ^a^	70.08 ± 0.03 ^a^	69.47 ± 0.06 ^a^
	C20:1n11	0.08 ± 0.00 ^a^	0.08 ± 0.01 ^a^	0.09 ± 0.01 ^a^
PUFA	C18:2n6t	19.73 ± 0.02 ^a^	19.17 ± 0.07 ^a^	19.85 ± 0.08 ^a^
	C18:3n6	0.04 ± 0.00 ^a^	0.04 ± 0.01 ^a^	0.05 ± 0.01 ^a^

Saturated fatty acids (SFA), monounsaturated fatty acids (MUFA) and polyunsaturated fatty acids (PUFA). Mean value ± standard deviation. The same letter in the same row indicates that there are no significant differences between the values (*p* > 0.05).

**Table 3:** Amino acid content per 100 g of protein in their three analysed forms: raw almonds (RA), peeled almonds (PA) and roasted almonds (RTA).

Amino Acids		RA (g/100 g Protein)	PA (g/100 g Protein)	RTA (g/100 g Protein)
**Essential**	**Ile**	3.92 ± 0.20 ^a^	3.78 ± 0.19 ^a^	3.75 ± 0.19 ^a^
	**Leu**	6.84 ± 0.34 ^a^	6.73 ± 0.34 ^a^	6.53 ± 0.33 ^a^
	**Lys**	3.50 ± 0.18 ^a^	3.40 ± 0.17 ^a^	3.22 ± 0.16 ^b^
	**Met + Cys**	2.61 ± 0.13 ^a^	2.60 ± 0.13 ^a^	2.47 ± 0.12 ^b^
	**Phe + Tyr**	8.72 ± 0.44 ^a^	8.61 ± 0.43 ^a^	8.35 ± 0.42 ^a^
	**Thr**	3.24 ± 0.16 ^a^	3.10 ± 0.17 ^a^	3.09 ± 0.15 ^a^
	**Trp**	1.39 ± 0.07 ^a^	1.34 ± 0.07 ^a^	1.23 ± 0.06 ^b^
	**Val**	5.30 ± 0.27 ^a^	5.19 ± 0.26 ^a^	5.09 ± 0.25 ^a^
**Non-essential**	**Asp**	11.52 ± 0.58 ^a^	11.39 ± 0.57 ^a^	11.17 ± 0.56 ^ab^
	**Glu**	19.49 ± 0.97 ^a^	19.31 ± 0.97 ^a^	19.01 ± 0.96 ^ab^
	**Arg**	11.53 ± 0.54 ^a^	11.27 ± 0.56 ^a^	11.16 ± 0.56 ^a^
	**Ala**	4.37 ± 0.22 ^a^	4.31 ± 0.22 ^a^	4.27 ± 0.21 ^a^
	**Gly**	4.25 ± 0.21 ^a^	4.12 ± 0.21 ^a^	4.07 ± 0.20 ^a^
	**Pro**	3.60 ± 0.16 ^a^	3.49 ± 0.17 ^a^	3.46 ± 0.17 ^a^
	**Ser**	4.58 ± 0.23 ^a^	4.51 ± 0.23 ^a^	4.37 ± 0.22 ^a^

Ile: Isoleucine; Leu: Leucine; Lys: Lysine; Met + Cys: Methionine + Cysteine; Phe + Tyr: Phenylalanine + Tyrosine; Thr: Threonine; Trp: Tryptophan; Val: Valine; Asp: Aspartic acid; Glu: Glutamic acid; Arg: Arginine; Ala: Alanine; Gly: Glycine; Pro: Proline and Ser: Serine. Mean value ± standard deviation. The same letter in the same row indicates that there are no significant differences between the values (*p* > 0.05).

## Data Availability

The original contributions presented in this study are included in the article. Further inquiries can be directed to the corresponding author.
